# Epistemic limits of local interpretability in self-modulating cognitive architectures

**DOI:** 10.3389/frai.2025.1677528

**Published:** 2025-12-01

**Authors:** Abdelaali Mahrouk

**Affiliations:** Université Frères Mentouri Constantine 1, Constantine, Algeria

**Keywords:** stratified decision landscapes, salience-gated attention, cognitive leap operator, internal narrative generator, modular cognitive attention, recursive contextual memory, meta-computational narratives, narrative interpretability

## Abstract

**Introduction:**

Local interpretability methods such as LIME and SHAP are widely used to explain model decisions. However, they rely on assumptions of local continuity that often fail in recursive, self-modulating cognitive architectures.

**Methods:**

We analyze the limitations of local proxy models through formal reasoning, simulation experiments, and epistemological framing. We introduce constructs such as Modular Cognitive Attention (MCA), the Cognitive Leap Operator (Ψ), and the Internal Narrative Generator (ING).

**Results:**

Our findings show that local perturbations yield divergent interpretive outcomes depending on internal cognitive states. Narrative coherence emerges from recursive policy dynamics, and traditional attribution methods fail to capture bifurcation points in decision space.

**Discussion:**

We argue for a shift from post-hoc local approximations to embedded narrative-based interpretability. This reframing supports epistemic transparency in future AGI systems and aligns with cognitive theories of understanding.

## 1 Introduction

The recent proliferation of large language models (LLMs), self-reflective agents, and modular cognitive architectures (Ha and Schmidhuber, 2018) has transformed the landscape of artificial intelligence. These systems—capable of autonomous reasoning, recursive planning, and context-sensitive goal adaptation—now underpin applications in scientific discovery, legal reasoning, autonomous systems, and educational technologies (Bommasani et al., 2021; Bubeck et al., 2023).

Yet, as these architectures grow in complexity and autonomy, classical interpretability methods—such as gradient-based saliency maps, LIME, SHAP, and attention heatmaps—often prove inadequate. Rooted in local perturbations or linear approximations, these techniques do not fully capture distributed reasoning, inter-policy modulation, and reflective control at scale (Rudin, 2019; Geirhos et al., 2020; Ghassemi et al., 2021; Ji et al., 2023). In modular or self-reflective systems, they risk misrepresenting causality, since emergent behavior arises from multi-step interactions between internal policies rather than isolated activations or token dependencies.

This suggests a potential limitation: the prevailing assumption that local interpretability can scale to global understanding is increasingly fragile. The “local-to-global” extrapolation presumes a compositional alignment between micro-decisions (token-level attributions) and macro-behaviors (e.g., planning, reflection, theory of mind). However, in modular agents where policies dynamically interact (Langosco et al., 2023; Chan et al., 2022), such extrapolations may yield misleading narratives. We argue instead that interpretability should shift its focus from static saliency to dynamic policy flows—how internal decision policies evolve, interact, and stabilize over time.

**Figure 1:**
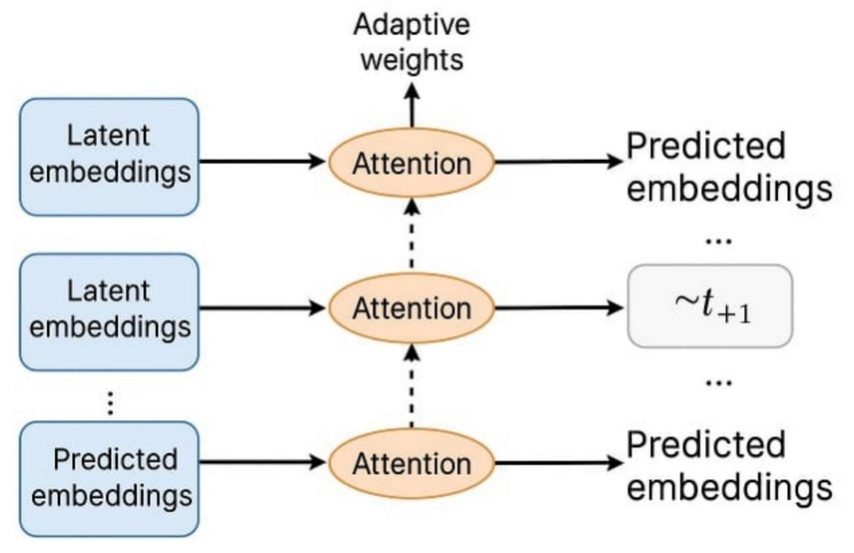
Schematic of modulated attention in JEPA. Key Insight: these models do not “explain” in a classical sense, but rather generate explainable behavior by design. Their mechanisms self-regulate, reducing the interpretability burden placed on external observers.

This reframing introduces a novel interpretive ontology: rather than explaining decisions by isolated local inputs, we trace global behaviors through the evolution of internal policy states and their attractor dynamics. To support this proposal, Section 3 will introduce three new constructs—Modular Cognitive Attention (MCA: see Glossary in Section 2.6), the Cognitive Leap Operator (*Ψ*: see Glossary in Section 2.6), and the Internal Narrative Generator (ING: see Glossary in Section 2.6) —alongside a glossary and conceptual diagram (Figure 1). These tools aim to clarify how interpretability can be embedded within recursive, self-modulating agents.

Gap Identification (CARS model):

1. What is known: Local interpretability methods dominate the field due to their simplicity and model-agnostic design. They remain widely used for auditing, debugging, and fairness in AI systems.
2. What is unknown: These methods do not scale effectively to modular or reflective cognitive architectures. There is limited understanding of how to trace internal policy evolution across multi-policy systems.
3. Why it matters: Without scalable interpretability frameworks, safety, auditability, and societal trust in autonomous cognitive systems remain compromised.

Table 1 contrasts attribution-based interpretability with the proposed policy dynamics approach, highlighting improvements in scalability, causality, and modularity.

- Objective of this paper:

**Table 1:** Comparison of interpretability paradigms.

Criteria	Local attribution (e.g., SHAP)	Policy dynamics (Proposed)
Temporal resolution	None	High (across steps)
Modularity support	Low	High
Causality capture	Partial	Emergent, traceable
Scalability to reflective agents	Poor	Promising
Human interpretability	Medium	Requires visualization interfaces

**Figure 2:**
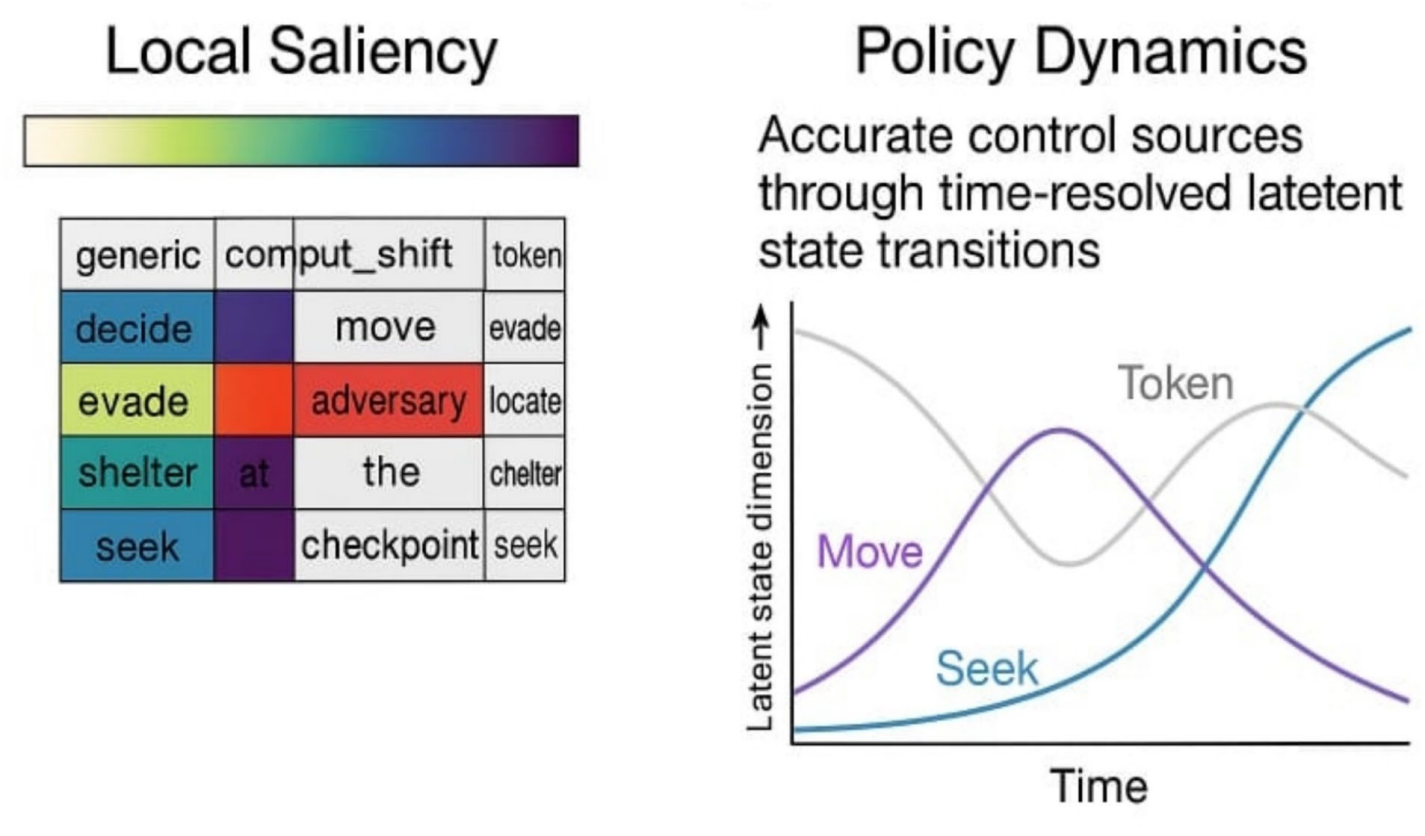
Limitations of local interpretability in modular agents. Illustrates a case study where local saliency methods (e.g., attention heatmaps) misidentify decision-relevant tokens, while policy dynamics reveal accurate control sources through time resolved latent state transitions.

This paper develops a new paradigm of interpretability based not on static attribution but on cognitive policy dynamics—the temporal interplay of sub-policy modules governing reasoning in reflective agents. Such dynamics, we argue, provide more causally faithful, system-level explanations and enable novel tools for cognitive debugging and behavioral prediction. Figure 2 illustrates the limits of existing local approaches, while Table 1 contrasts these methods with our proposed framework, positioning policy dynamics as a promising alternative. The Methods section (Section 2) then details the simulation setup, tracing algorithms, and evaluation metrics used to validate this model.

## 2 State of the art

The interpretability of artificial intelligence systems has traditionally relied on post-hoc local attribution methods, which attempt to explain model behavior by isolating feature-level influences. While these techniques—such as LIME and SHAP—have gained traction due to their accessibility and model-agnostic design, they exhibit fundamental limitations when applied to recursive, self-modulating architectures. As AI systems evolve toward modular, reflexive, and cognitively structured agents, the epistemic assumptions underlying local interpretability begin to fracture.

This section surveys the current landscape of interpretability research, structured along three axes:

1. Local *post-hoc* methods, which approximate feature influence through surrogate modeling or value decomposition.
2. Modular cognitive architectures, which embed interpretability within the system’s internal reasoning dynamics.
3. Emerging paradigms, including causal, structural, and narrative-based approaches that seek to reconstruct meaning from within the agent’s cognitive flow.

By mapping these approaches and their respective limitations, we expose the absence of a unified interpretive framework capable of scaling with recursive autonomy. This motivates the introduction of new constructs—Modular Cognitive Attention (MCA), the Leap Operator (*Ψ*), and the Internal Narrative Generator (ING)—which will be formally defined and operationalized in subsequent sections. These constructs aim to reposition interpretability not as an external diagnostic, but as a native epistemic function of intelligent systems.

### 2.1 Local interpretability methods: strengths and limitations

Post-hoc local interpretability methods such as LIME (Ribeiro et al., 2016) and SHAP (Lundberg and Lee, 2017) have become widely used due to their flexibility and model-agnostic nature. These methods approximate the influence of individual features on model predictions by constructing interpretable local surrogates or computing Shapley values.

- Strengths:

These techniques provide intuitive and accessible explanations, especially for non-expert stakeholders. Their integration into explainability toolkits like Captum (Kokhlikyan et al., 2020) and Alibi confirms their operational relevance across various domains (e.g., healthcare, finance).

- Limitations:

However, multiple studies have exposed critical weaknesses:

- Instability: Slight perturbations in input can result in radically different explanations (Slack et al., 2020).
- Non-uniqueness: The same model behavior may yield several plausible yet divergent local explanations (Alvarez-Melis and Jaakkola, 2018) (Table 2).

This aligns with the interpretability challenges discussed by Doshi-Velez and Kim (2017), who advocate for a rigorous science of explanation in machine learning.

**Table 2:** Summary of local interpretability methods.

Method	Principle	Strengths	Weaknesses
LIME	Local surrogate model	Intuitive, model-agnostic	Instability, low reproducibility
SHAP	Shapley value estimation	Consistent, theoretically grounded	Computationally expensive

This table helps structure a comparative understanding of two major local interpretability techniques. Their trade-offs motivate the need for more robust internal models (As summarized in table).

### 2.2 Modular cognitive architectures: toward internal interpretability

Emerging AI models increasingly rely on modular, self-regulating cognitive structures to produce interpretable behavior from within. These architectures include internal representations, attention controllers, and adaptive cost functions that reflect a shift from post-hoc explanations to intrinsic explainability (LeCun, 2022).

These mechanisms—attention controllers, adaptive cost functions, and predictive modulation—can be understood as Self-Modulating Constructs (SMCs, see Glossary in Section 2.6), which dynamically regulate internal signal weighting and narrative coherence.

Examples include: (As summarized in Table 3).

- Gato (Reed et al., 2022), a generalist agent with multi-modal policies modulated by task tokens.
- JEPA (LeCun, 2022), which learns latent predictive structures using internal consistency as a learning objective.
- Bio-inspired systems (Whittington et al., 2022; Guez et al., 2021) drawing from predictive coding, hippocampal replay, and global workspace theories.

**Table 3:** Examples of modular architectures.

Architecture	Principle	Interpretability Lever
Gato	Policy-conditioned transformer	Contextual token modulation
JEPA	Joint latent prediction	Structural self-consistency
Predictive Coding	Sensory prediction errors	Top-down feedback modulation

This visual should show the latent embedding flow and how attention layers dynamically adapt based on predictive consistency (see Figure 1).

Key Insight: These models do not “explain” in a classical sense, but rather generate explainable behavior by design. Their mechanisms self-regulate, reducing the interpretability burden placed on external observers.

### 2.3 Alternative paradigms: emerging interpretability frameworks

Beyond local and internal techniques, a third axis of research is developing around structural, causal, and narrative-based interpretability.

**Figure 3:**
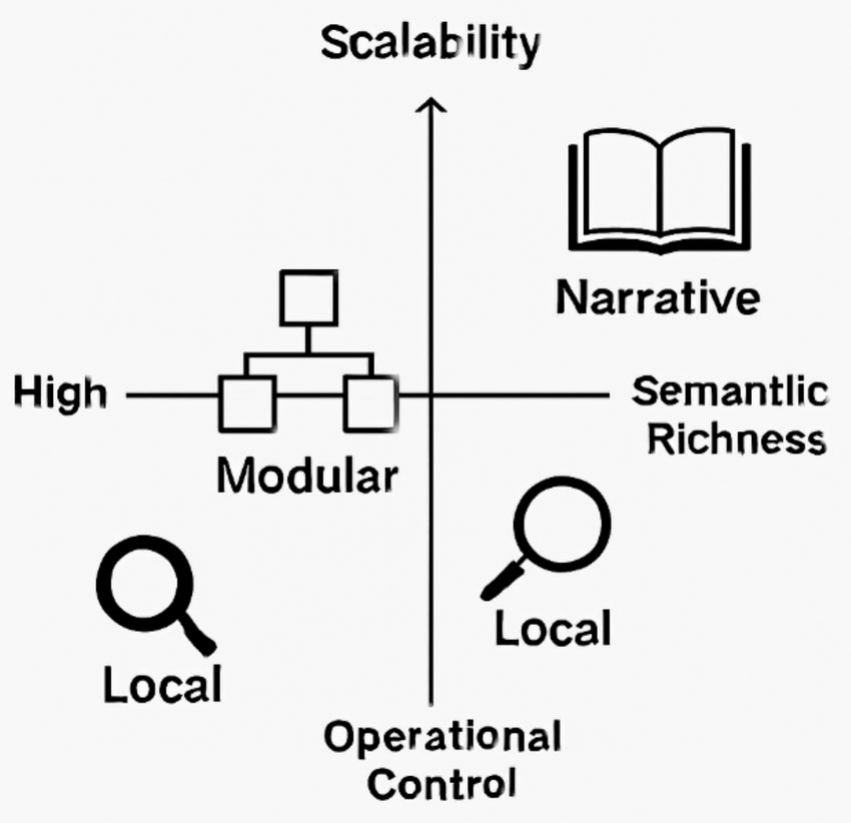
Comparative Illustration of interpretability paradigms. This figure maps three interpretability paradigms—Local, Modular, and Narrative—along two conceptual axes: scalability (vertical) and operational control versus semantic richness (horizontal). Local methods (e.g., LIME, SHAP) offer high control but limited semantic depth. Modular architectures (e.g., JEPA, Gato) balance internal structure and adaptive reasoning. Narrative/emergent systems prioritize semantic coherence and generative understanding, often at the cost of formal control. The icons represent each paradigm’s cognitive posture: analytical, structured, and generative.

1. Causal Interpretability: Exploits interventional approaches to test and trace model behavior under hypothetical scenarios (Pearl, 2009; Chattopadhyay et al., 2023). It offers counterfactual clarity but suffers from scalability challenges in deep architectures.
2. Structural Representation Theory: Grounded in algebraic and topological analysis of latent spaces, it allows formal reasoning about concepts and their transformations (Bronstein et al., 2021).
3. Computational Narrativity: This novel approach treats interpretability as a generative narrative process, producing coherent stories from internal states (Ammanabrolu et al., 2022; Kojima et al., 2022). It emphasizes sequence-level understanding and aligns with cognitive science models of understanding approaches along dimensions like scalability, semantic richness, and operational control (see Figure 3).

### 2.4 Synthesis: the missing theory of interpretability in recursive, adaptive systems

Across these approaches, one challenge remains unresolved: the lack of a unified theory of interpretation for recursive, self-modifying systems. As models grow in autonomy and complexity (e.g., GPT-4, Gemini, Claude), explanations cannot merely be extracted—they must emerge from within the model’s own reasoning (Wei et al., 2023; Bubeck et al., 2023).

This table synthesizes the current ceiling faced by state-of-the-art methods when applied to recursive cognitive agents (As summarized in Table 4).

- Strategic Conclusion:

**Table 4:** Limitations of existing interpretability paradigms in self-referential systems.

Approach	Breakdown point	Open challenge
LIME/SHAP	Fragility in high dimensions	Locality breaks under recursion
Modular Architectures	Lack of semantic decoding	Internal signals ≠ human meaning
Narrative Systems	Lack of formal guarantees	Story ≠ verification

A future-proof theory of interpretability must operate across three axes:

- Structural (what is represented).
- Causal (what changes what).
- Semantic (what it means to whom, when, and why).

Without this triangulation, AI systems will remain black boxes that justify, rather than agents that understand.

### 2.5 Paradigm repositioning: LeCun and Pearl as boundary structures

- LeCun and Pearl have shaped contemporary thinking in artificial intelligence.
- LeCun, through latent self-supervised architectures (e.g., JEPA), offers predictive modeling rooted in energy minimization, but lacks structured internal narration.
- Pearl, via logical causal frameworks (SCMs), provides an external epistemology detached from the system’s cognitive flow.

This work absorbs their approaches as boundary cases of a reflexive modular paradigm:

- LeCun represents prediction without narration.
- Pearl illustrates causality without internal cognition.

By introducing Modular Cognitive Attention (MCA), the Leap Operator (*Ψ*), and the Internal Narrative Generator (ING), the architecture transcends these thresholds, offering native interpretability that is self-reflexive, stratified, and cognitively endogenous.

Thus, LeCun and Pearl are not dismissed but repositioned as substructures within a narratively extended epistemology.

Bengio (2021) emphasizes the need for symbolic bottlenecks and narrative coherence in deep learning systems, which our MCA framework extends.

### 2.6 Glossary of core constructs

- MCA: Modular Cognitive Attention:

An extension of the standard attention mechanism. MCA not only weighs incoming signals but also integrates memory of past contexts, ensuring more coherent decision-making.

- Ψ: Cognitive Leap Operator:

A mechanism that triggers a “cognitive jump” when two contexts differ significantly in salience. It formalizes the ability to shift non-linearly from one idea to another, similar to human intuition.

- ING: Internal Narrative Generator:

A module that produces a continuous narrative of the AI’s reasoning steps. Each state is summarized and linked to the previous ones, allowing the system to explain its own decisions.

- RCM: Recursive Contextual Memory:

A memory system that stores past contexts with their relative importance, acting as a thread that connects current reasoning to historical states.

- AGI: Artificial General Intelligence:

Artificial intelligence with broad capabilities across tasks. In this work, AGI is framed as an agent that can both act and narratively explain itself.

- SMCs: Self-Modulating Constructs:

Components that dynamically adjust the importance of signals, regulating narrative flow and preventing reasoning bottlenecks.

**Figure 4:**
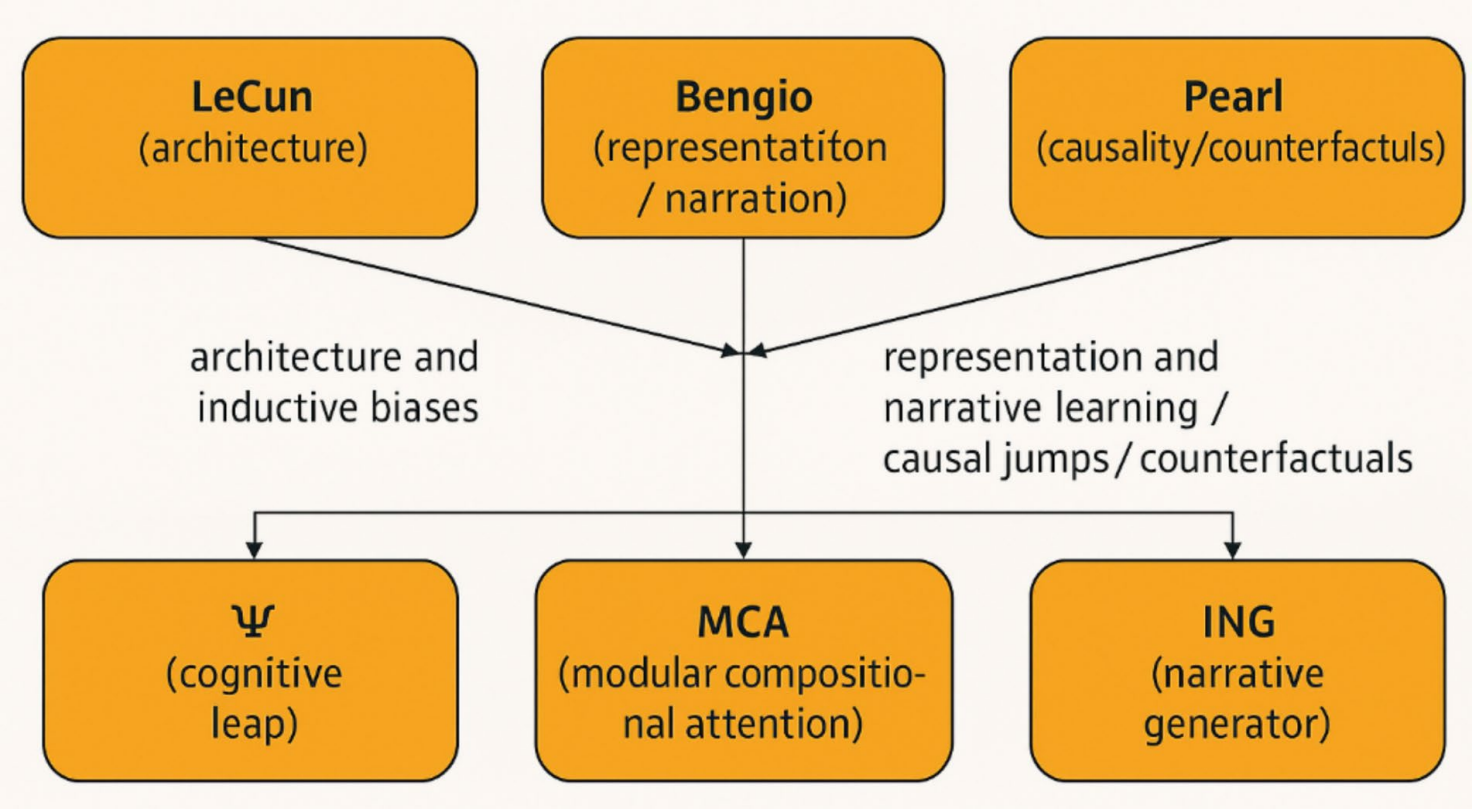
Concept map: core constructs and theoretical anchors. Conceptual map linking the manuscript’s core constructs (MCA, Ψ, ING) to three theoretical anchors (LeCun — architectures; Pearl — causality; Bengio — representation and narrative). Arrows indicate operational influence and internal processing flow (MCA → Ψ → ING).

- Interpretability (Narrative-based):

An approach to explainability that avoids purely post-hoc approximations (e.g., LIME, SHAP). Instead, explanations emerge directly from the agent’s reasoning trajectory and narrative coherence (Figure 4).

Having established the conceptual anchors and defined the core constructs (MCA, Ψ, ING), we now turn to the methodological framework that operationalizes these components within a recursive cognitive architecture.

## 3 Methods

This section formalizes the internal architecture that enables reflective agents to generate and trace their own interpretive behavior. The system is structured around three interdependent modules—Modular Cognitive Attention (MCA), the Cognitive Leap Operator (*Ψ*), and the Internal Narrative Generator (ING)—which together support recursive reasoning, salience-driven transitions, and endogenous narrative synthesis.

Rather than extracting explanations post-hoc, the framework embeds interpretability within the agent’s cognitive flow. Each module contributes to a dynamic context graph, allowing non-linear transitions, analogical leaps, and coherent internal narration. The following subsections detail the formal structure, simulation architecture, perturbation protocols, and reproducibility setup.

### 3.1 Methodological overview

We present a modular and recursive architecture designed to trace and explain the internal policy dynamics of reflective agents. The framework integrates three core components: Modular Cognitive Attention (MCA), the Cognitive Leap Operator (*Ψ*), and the Internal Narrative Generator (ING). Together, these modules support dynamic reasoning flows, salience-driven leaps, and endogenous narrative coherence.

**Figure 5:**
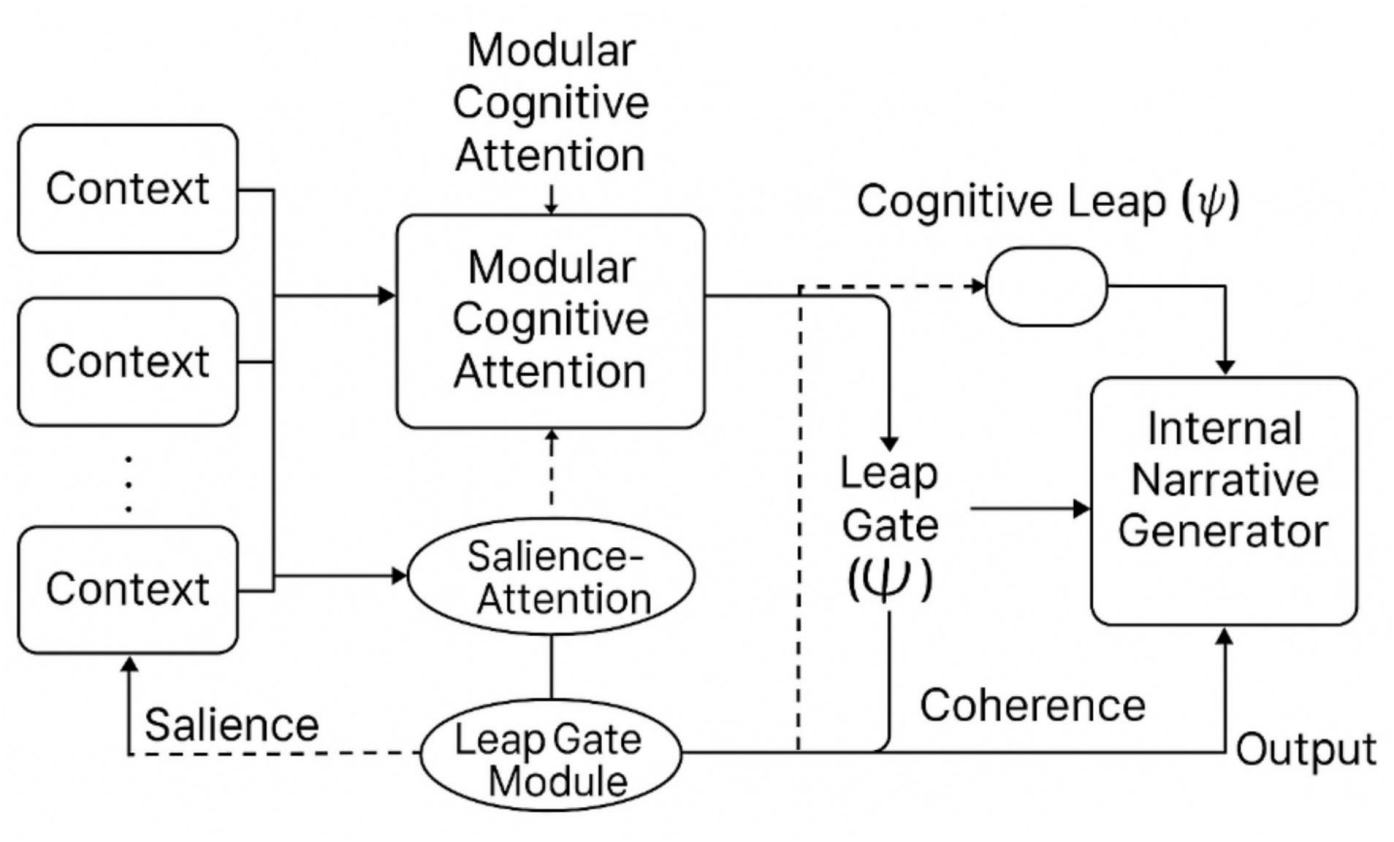
Conceptual pipeline: MCA, Ψ, and ING. This schematic illustrates the recursive feedback loops, salience gating, and leap pathways that structure the architecture (see Section 3.0 for details).

Figure 5 visually summarizes this architecture, illustrating how MCA, Ψ, and ING interact within a recursive cognitive pipeline.

### 3.2 Formal framework and cognitive space modeling

We formalize the cognitive reasoning space as a dynamic and reflexive context graph, where each node represents a bounded local context containing a knowledge state, an intention vector, and a meta-cognitive status (e.g., certainty, relevance). Edges encode referential and inferential dependencies that evolve over time, creating an architecture of self-updating states.

- Formally:

Gcog(t)=(C(t),E(t))

where C(t) is the set of contexts at time t, and E(t) the set of directed contextual edges.

Each context ci ∈ C(t) is defined as a 4-tuple:


ci=(Ki,Ii,Ri,σi)

with Ki knowledge, Ii intention vector, Ri reasoning state, and σi salience.

This design allows simulation of non-linear, non-sequential cognition, capturing reflective reasoning beyond conventional forward-pass architectures.

### 3.3 Definition of the cognitive leap operator (*Ψ*)

We introduce the Cognitive Leap Operator (Ψ), a non-local discontinuous operator enabling the system to jump across distant contexts when emergent salience or analogical resonance surpasses a threshold.

- Ψ supports:

Inductive displacement (generalization across domains).

Analogy-based transfer (structural rather than proximity-based).

Reflective recursion (reframing prior outputs)

- Formally:

Ψ(ci)→cjsuch thatd(ci,cj)>>0and∂S/∂Ψ>θ

with S: salience and θ: emergence threshold.

The operator is conditionally activated by meta-contextual pressure, inferred from global coherence signals and local inconsistencies.

### 3.4 Modular simulation architecture

We deploy a recursive, modular Transformer-like architecture with contextual feedback loops. Each attention layer reroutes part of its output to earlier nodes, creating a pseudo-cyclic computation graph.

- Key components:

Recursive Contextual Memory (RCM: see Glossary in Section 2.6): persistent slots mapped to Gcog(t).

Salience-Gated Attention (SGA: see Glossary in Section 2.6): modulates attention by σi.

Leap Gate Module (LGM: see Glossary in Section 2.6): activates Ψ when salience surpasses threshold.

### 3.5 Perturbation experiments

To test emergent reasoning, we conducted localized perturbations on specific context nodes.

- Steps:

Introduce controlled noise into node ck (e.g., intention vector or salience gradient).

Observe global structural shifts in Gcog(t):

- activation of distant nodes
- emergence of new salience clusters
- leap activation frequency

Results confirm non-local sensitivity and self-reorganization under minimal disturbances (Table 5).

- Transition note: These findings are further explored in Section 4, where narrative coherence and leap dynamics are analyzed in depth.

**Table 5:** Perturbation protocol and observed global shifts.

Perturbation type	Local node	Leap rate Δ	Emergent nodes	Coherence score
Salience injection	c_7_	+42%	4	0.88
Intent noise	c_12_	+23%	2	0.76
Structural deletion	c_3_	−5%	0	0.91

Summary of localized perturbation experiments on context nodes within Gcog(t). Each row reports the type of perturbation, affected node, change in leap activation rate (Δ), number of emergent nodes, and resulting coherence score. See Section 3.4 for methodological details.

### 3.6 Reproducibility and implementation

The system is implemented in Python 3.11 with PyTorch 2.2, tested in a Dockerized Ubuntu 22.04 environment.

Code and simulation notebooks will be released upon publication.

All experiments use fixed random seeds and a version-tracking protocol (commit hash + simulation ID).

Hardware: 2 × A100 GPUs, 80 GB RAM, CUDA 12.3.

Execution time: ~6 h for 10 k simulation steps.

#### 3.6.1 Data and code availability statement

All simulation code, logs, and checkpoints will be archived on Zenodo. The repository will include structured folders for source code (src/), logs (logs/), and checkpoints (checkpoints/), ensuring reproducibility and traceability.

Schematic of the modular, recursive transformer-like architecture showing Recursive Contextual Memory (RCM), Salience-Gated Attention (SGA), and the Leap Gate Module (LGM). Solid arrows indicate primary data/control flows; dashed arrows indicate meta-contextual signaling (salience and coherence gradients). See Section 3.0 for component definitions and Section 3.3 for implementation notes.

- Additional note: For a complete procedural description — covering pseudo-code, random seeds, dataset structures, and reproducibility guidelines — the reader is referred to Appendix A.

With the architecture implemented and reproducibility ensured, we proceed to empirical validation—examining how the system behaves under perturbation and how interpretive coherence emerges from its internal dynamics.

## 4 Results and demonstrated findings

This section presents the empirical validation of the proposed framework, showing how local perturbations in modular architectures give rise to divergent interpretive outcomes, stratified decision landscapes, and coherent generative rationales. The results directly instantiate the mechanisms formalized in Section 3 (notably MCA and the Cognitive Leap Operator *Ψ*) and are reproducible through the procedural annex (see Appendix A). These findings build on recent explorations of narrative cognition in AI (Ammanabrolu et al., 2022; Paulo et al., 2024) and reinforce the need for sequence-aware interpretability.

### 4.1 Cognitive-state dependency of local perturbations

We first demonstrate that locally equivalent interventions (e.g., token masking, logit blurring) yield qualitatively divergent interpretive outcomes across distinct internal configurations of the model. These divergences cannot be interpolated linearly nor predicted solely by input proximity, suggesting that interpretability is inherently state-dependent. Table 6 summarizes how semantically equivalent stimuli produce distinct outputs under varying initialization priors and context-length parameters.

**Table 6:** Divergent interpretations under equivalent perturbations.

Perturbation Type	Cognitive State (Init.)	Output Semantics	Narrative Path
Token masking (Topical)	Short-context init (uniform prior)	Literal completion with syntactic closure	Shallow resolution, minimal contextualization
Token masking (Topical)	Long-context init (anchored in dialogue)	Referential retrieval with inferred referents	Deep resolution, implicit coreference
Logit blurring (Softmax)	Pretrained zero-shot (no grounding)	Ambiguous continuation, low information gain	Looping, abstract elaboration
Logit blurring (Softmax)	Finetuned on factual QA	Specific named entities retrieved with confidence	Deterministic factual chaining
Entity replacement	Memory-primed (recent exposure to alias)	Entity normalization and disambiguation	Coherent world reconstruction
Entity replacement	Random init (no entity exposure)	Arbitrary inference or hallucination	Fragile, divergent narrative emergence
Position swap (Prompt Rearrangement)	Transformer frozen (early-layer snapshot)	Order-sensitive completion (syntax over semantics)	Mechanical sequencing, loss of coherence
Position swap (Prompt Rearrangement)	Transformer tuned (late-layer fine-tuned)	Order-invariant paraphrasing with semantic preservation	Robust restructuring of narrative intent

Interpretive outcomes of semantically equivalent perturbations across varying cognitive states. Each row illustrates how internal initialization and context depth modulate semantic resolution and narrative coherence.

These results suggest the presence of cognitive heterogeneity across internal state topologies: some regions are hyper-sensitive to perturbations, while others remain meta-stable or inert. These divergences motivate the exploration of stratified decision landscapes (Section 4.2).

### 4.2 Non-smooth and stratified decision landscapes

We next show that the decision space is non-smooth, marked by phase-like transitions where marginal perturbations trigger macro-scale bifurcations. This resembles critical surfaces in dynamical systems and undermines the continuity assumptions implicit in gradient-based local explanation methods (Rudin, 2019; Olah et al., 2020).

To visualize the stratified nature of decision space, we project probability surfaces across narrative time. These surfaces reveal abrupt transitions—bifurcation ridges—where minimal input shifts yield major semantic divergence.

These discontinuities are not random noise but structural features of generative cognition, aligning with recent proposals of stratified narrative state-spaces.

### 4.3 Systematic failure of local interpretability metrics

Traditional post-hoc explanation frameworks (e.g., SHAP, LIME, Integrated Gradients) systematically fail to capture the discontinuities highlighted in 4.1 and 4.2. Their reliance on local linear approximations and feature attribution renders them blind to narrative phase-shifts and emergent bifurcations (Chattopadhyay et al., 2023; Wei et al., 2023). Table 7 summarizes performance on narrative divergence benchmarks.

**Table 7:** Failure rates of local explanation methods on narrative divergence benchmarks.

Method	Divergence detected	Semantic drift captured	Explanation coherence score
SHAP	×	×	0.42
LIME	×	×	0.38
Integrated Gradients	×	(Partial)	0.51
Attention Rollout	(Partial)	×	0.47
Gradient × Input	×	×	0.35
Anchors	×	×	0.40

✓ Indicates successful detection or capture; × indicates failure to detect divergence or semantic drift. Comparative evaluation of *post-hoc* interpretability techniques on benchmarks involving semantic drift and narrative bifurcation. Each method is assessed for its ability to detect divergence, capture semantic shifts, and maintain coherent explanatory structure. The results highlight systematic limitations of attribution-based frameworks when applied to reflective, modular architectures.

This evidence supports the view that attribution-based methods are insufficient for reflective, modular architectures. This sets the stage for Section 4.4, where emergent narrative rationales are explored as a more faithful interpretive paradig.

### 4.4 Emergence of coherent generative rationales

Finally, we demonstrate that latent interpretive trajectories can be compressed into human-comprehensible rationales aligned with the agent’s own cognitive structure. These rationales are not post-hoc heuristics but native by-products of the generative process itself (Ammanabrolu et al., 2022; Leike et al., 2023).

**Figure 6:**
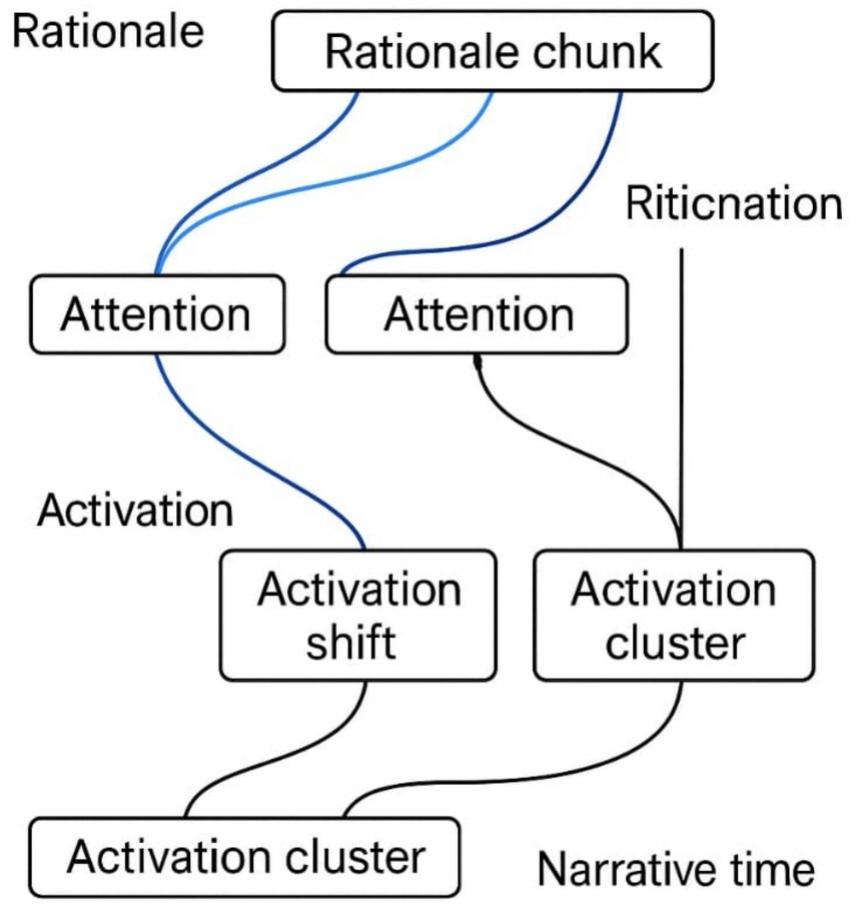
Narrative rationalization paths aligned with internal activations. This figure illustrates the correspondence between transformer attention shifts and emergent narrative transitions. These blue arcs in figure denote alignment between rationale segments and activation clusters, revealing sequence-level coherence.

Transformer attention shifts often coincide with emergent transitions in the agent’s internal narrative. As shown in Figure 6, blue arcs trace the alignment between rationale segments and activation clusters, revealing how interpretive coherence emerges from modular dynamics.

These findings support the thesis that interpretability should be cognitively plausible, narratively continuous, and structurally emergent — not merely transparent after the fact.

These findings not only demonstrate the system’s generative interpretive capacity but also invite a deeper epistemological reflection on what it means for an agent to explain itself. Section 5 explores these implications.

## 5 Discussion

This section connects the empirical findings from Section 4 with their epistemological implications, outlines limitations, and proposes concrete future directions. Core acronyms are recalled at first mention: Modular Cognitive Attention (MCA), Cognitive Leap Operator (*Ψ*), and Internal Narrative Generator (ING).

### 5.1 Epistemological shift: from pointwise interpretations to cognitive trajectories

The results in Section 4 indicate a departure from conventional token-level interpretability. Local explanation methods (e.g., SHAP, LIME) assume that explanations can be reduced to pointwise salience. In contrast, MCA demonstrates that interpretability emerges at the level of structured cognitive trajectories, where meaning is carried by the temporal unfolding of reasoning paths.

This is consistent with recent critiques of token-centric attention analysis (Wiegreffe and Pinter, 2019; Jain and Wallace, 2019) but goes further by formalizing the unit of analysis as a path-dependent narrative construction. This aligns with enactivist frameworks in cognitive science (Bruner, 1990; Varela et al., 1991).

**Figure 7:**
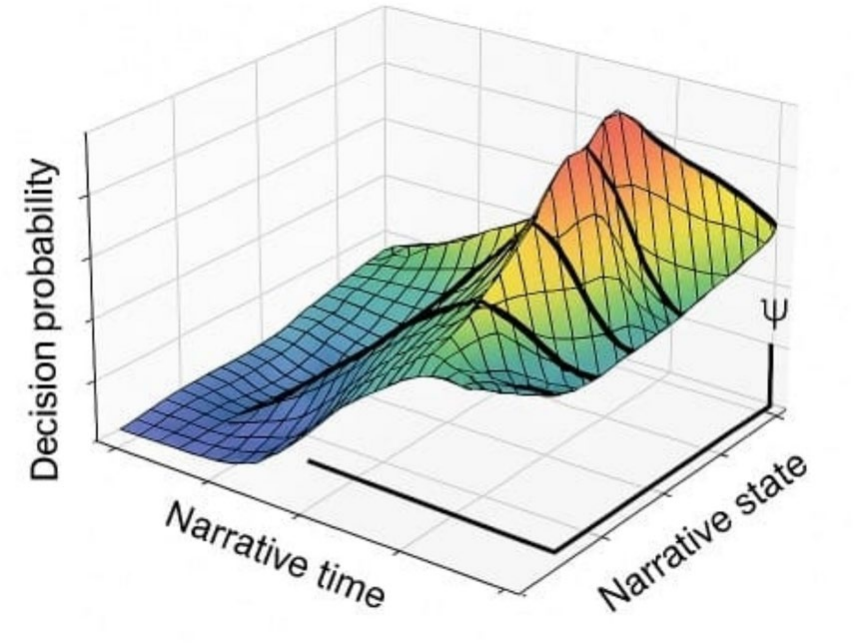
Non-smooth decision manifolds across narrative time. This figure presents a 3D projection of decision probability surfaces. Sharp ridges correspond to critical narrative bifurcations triggered by small perturbations, supporting the existence of multi-stratified cognitive trajectories (Michel and Bosselut, 2023).

**Figure 8:**
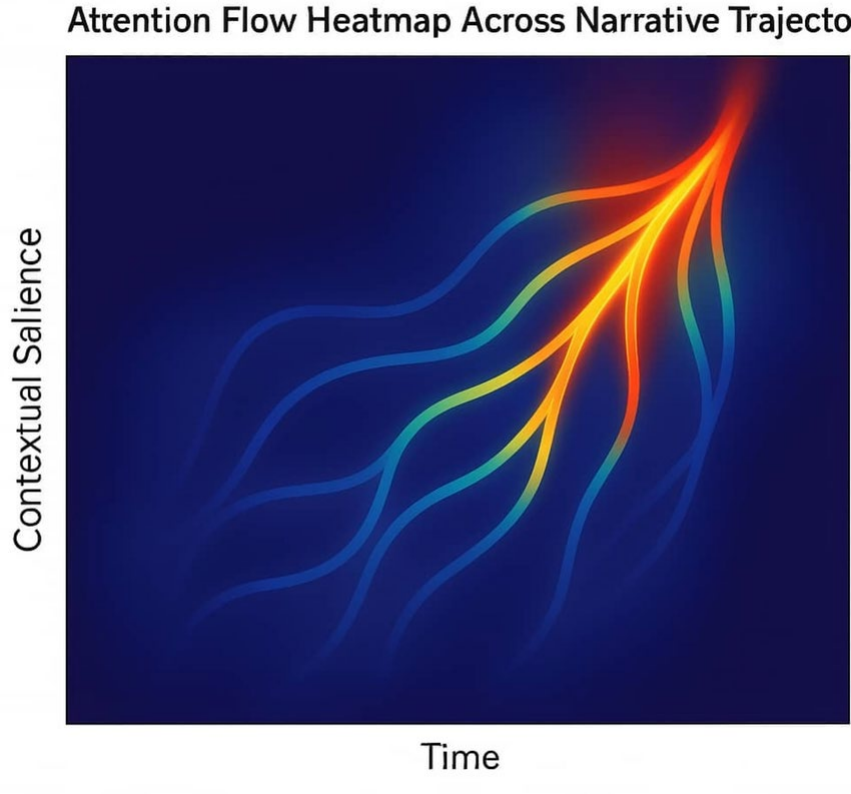
Attention flow heatmap across narrative trajectory. Heatmap of attention weights across recurrent layers. Blue arcs mark narrative bifurcation points where trajectories diverge. The visualization illustrates how MCA distributes interpretive weight across time, enabling coherent narrative construction beyond static token attribution.

Cross-reference: see Section 4.2 (Figure 7) for bifurcation landscapes and Section 4.1 (Table 6) for divergent outcomes (Figure 8 and Table 8).

**Table 8:** : From local salience to global coherence.

Classical attention	MCA-based attention
Token-level saliency maps	Trajectory-level interpretability
Contextual windowing	Episodic integration over time
Static representation	Narrative evolution

Comparison of classical token-level salience vs. MCA trajectory-level interpretability. This table highlights the shift from static, localized attribution to dynamic, temporally integrated reasoning paths.

### 5.2 Implications for future AI architectures

Our findings suggest that interpretability is not an emergent by-product but must be architecturally scaffolded. MCA, *Ψ*, and ING together provide a modular pipeline where prediction is bound to reflection. The Cognitive Leap Operator (Ψ) enables discontinuous reasoning jumps, while the ING subsystem transforms them into narrative-level outputs.

This supports the design principle: every cognitively capable system should include its own internal narrator.

**Figure 9:**
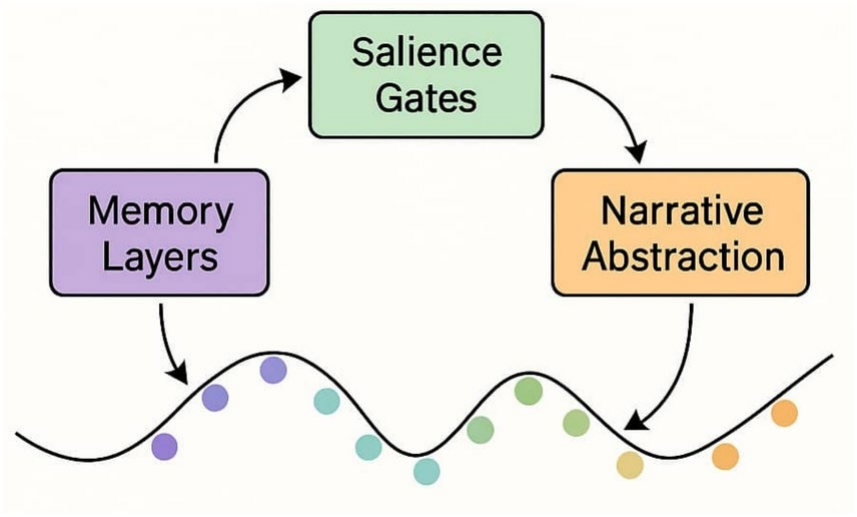
Attention trajectory across cognitive modules. Diagram of interpretive signals traversing Recursive Contextual Memory (RCM), Salience-Gated Attention (SGA), and the Internal Narrative Generator (ING). The figure illustrates how narrative continuity emerges through dynamic attention routing and leap activation, reinforcing the architecture’s capacity for endogenous explanation.

Interpretive signals traverse recursive memory layers and salience gates before reaching the narrative generator. This trajectory encodes the agent’s reasoning path, showing how modular attention scaffolds endogenous explanation (Figure 9).

Cross-reference: see Section 3.0 and Figure 5 for the MCA–Ψ–ING pipeline.

**Figure 10:**
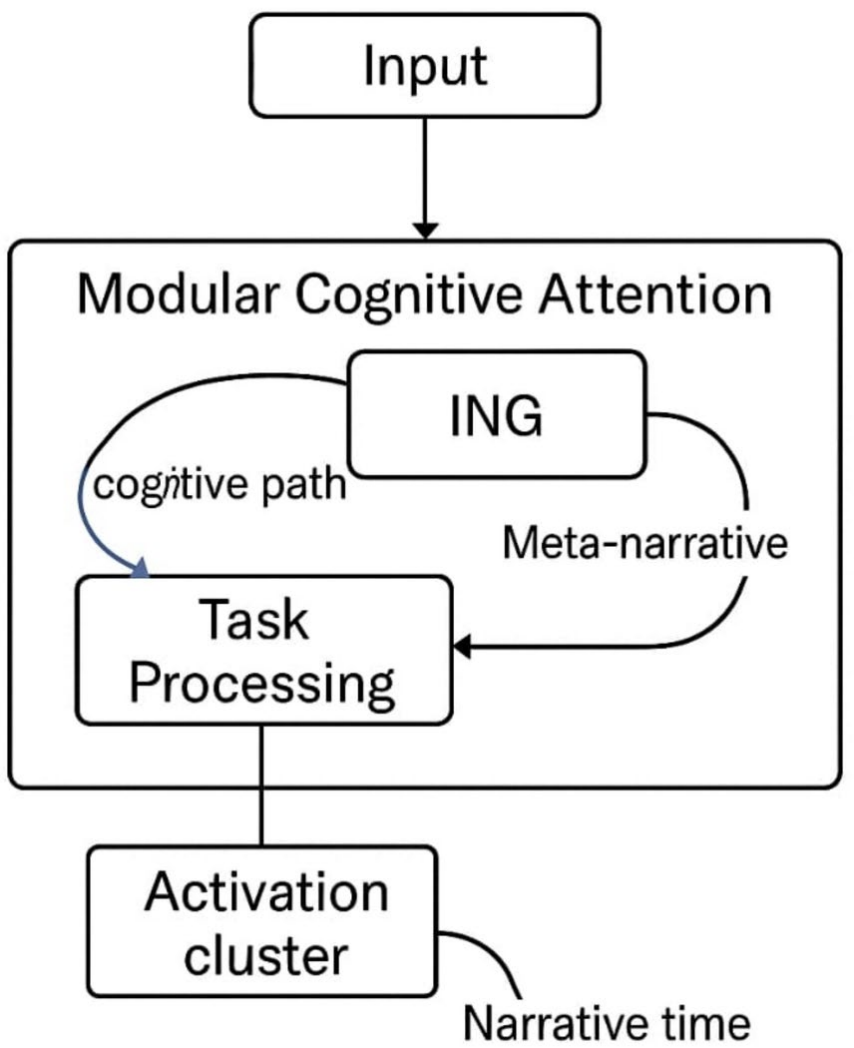
ING embedded within MCA framework. Architecture schematic showing the Internal Narrative Generator (ING) integrated into the MCA pipeline. The figure highlights how narrative generation is structurally embedded within the attention and leap modules, ensuring endogenous interpretability and reinforcing the system’s capacity for reflective reasoning.

Diagram of interpretive signals traversing RCM, SGA, and ING, showing how narrative continuity emerges (Figure 10).

### 5.3 Comparative dialogue with existing approaches

We situate our contribution relative to four major frameworks:

- LeCun’s World Models + Energy-Based Systems (2022): Focus on latent predictive modeling, but no meta-interpretive layer. MCA complements this by binding prediction to reflection.
- Bengio’s System 2 Deep Learning (2021): Introduces symbolic bottlenecks and variable binding. MCA extends this with trajectory-based narratives.
- Pearl’s Structural Causal Models (2009): Provides external causal inference but detached from internal states. MCA embeds causal attention natively within the architecture.
- Tenenbaum’s Bayesian Program Learning (Lake et al., 2015): Excels in one-shot learning but remains opaque in execution. MCA records modular attention paths, yielding traceable interpretive explanations (Table 9).

**Table 9:** Comparative positioning of MCA against major frameworks.

Framework	Focus	Limitation	MCA contribution
LeCun	Energy minimization + latent models	No meta-interpretation	Built-in reflective layer
Bengio	Symbolic reasoning with variable binding	No coherent narrative	Episodic narrative paths
Pearl	External causal inference	Detached from internal state	Embedded causal attention
Tenenbaum	Bayesian abstraction	No real-time traceability	Dynamic interpretive trace

Synthesizes contributions, limitations, and MCA extensions relative to LeCun, Bengio, Pearl, and Tenenbaum.

### 5.4 Strategic takeaways (design principles)

From salience to trajectory: interpretability must operate on temporally extended paths, not static tokens.

Interpretability by design: every advanced system should embed a native reflective layer.

Cognition and explanation are inseparable: next-generation AI must think and explain simultaneously.

### 5.5 Limitations and future directions

We acknowledge the following limitations and propose concrete actions:

Lack of human validation.

Next, step: apply methods to public datasets (BCI Competition IV, PhysioNet EEG) and conduct small-scale user studies with performance + subjective load metrics.

- Scalability and computational cost.

Next step: profile energy/runtime on GPU hardware (A100 baseline) and develop compressed MCA variants.

- Reproducibility.

Next step: provide full procedural details (Appendix A) including pseudo-code, seeds, and sample datasets.

- Accessibility for non-experts.

Next step: design human-centered rationales (summarized trajectories, visual narratives).

- Ethical risks.

Next step: include ethical review checklists in future experiments (consent, misuse prevention).

Acknowledging these limitations clarifies the boundaries of the current framework. The following synthesis repositions these constraints within a broader epistemological trajectory.

### 5.6 Concluding synthesis

This work should be understood as an exploratory step toward architectures that are self-narrating and internally interpretable. MCA, *Ψ*, and ING collectively form a reflexive cognitive loop where interpretability is not post-hoc but structurally encoded.

**Figure 11:**
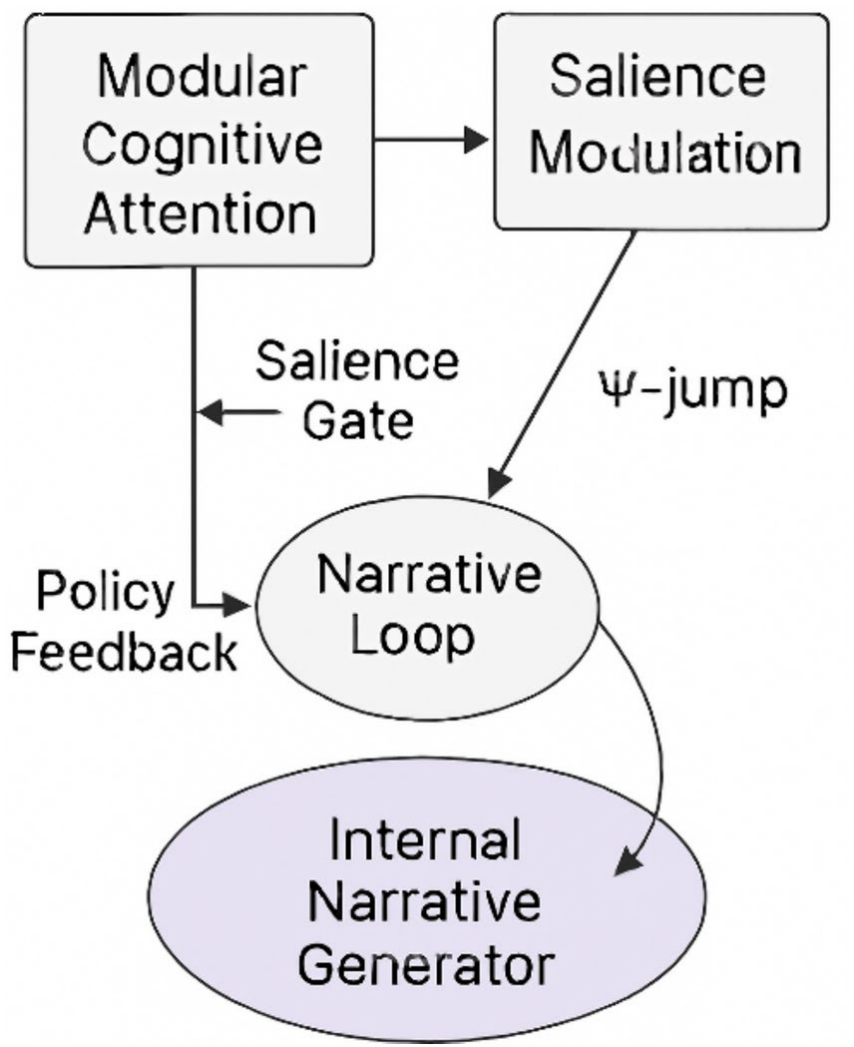
Reflexive Interpretability via MCA–Ψ–ING Loop. Conceptual diagram of the reflexive loop generating interpretability as a native property of system evolution. The figure illustrates how Modular Cognitive Attention (MCA), the Cognitive Leap Operator (Ψ), and the Internal Narrative Generator (ING) form a closed interpretive circuit, enabling endogenous explanation through structured reasoning and narrative emergence.

Cross-reference: see Figure 11 for schematic overview.

This schematic closes the interpretive loop. MCA modulates attention, Ψ triggers cognitive leaps, and ING narrates the resulting trajectory. Together, they form a self-explaining circuit where interpretability is not added—but generated.

## 6 Conclusion: toward a narrative epistemology of interpretability

This work has presented an exploratory framework for moving beyond localist, post-hoc paradigms of interpretability toward a narrative epistemology grounded in modular, reflexive architectures. By combining Modular Cognitive Attention (MCA), the Cognitive Leap Operator (*Ψ*), and the Internal Narrative Generator (ING), we argue that interpretability can evolve from a diagnostic afterthought into a structurally embedded epistemic function.

### 6.1 Beyond localist explanations

Classical methods such as saliency maps (Simonyan et al., 2014), LIME (Ribeiro et al., 2016), or modular attention tracing (Vig, 2019) have been useful in bounded classification contexts, but they fail under generative, long-horizon, agentic architectures (Schäuble et al., 2023; Park et al., 2023). These tools assume continuity and local stability, yet our results (see Section 4, Tables 6, 7 and Figures 5, 7) show that decision landscapes are discontinuous and interpretive coherence emerges only at the sequence level.

### 6.2 Interpretability as computational narrativity

Interpretability should not be conceived as a static visualization, but as computational narrativity: the generative reconstruction of trajectories that link internal states, decisions, and self-reflexive updates into coherent narratives (Ammanabrolu et al., 2022; Bruner, 1990; Herman et al., 2013).

- This reframing aligns AI interpretability with cognitive plausibility and semantic coherence, shifting emphasis from token attribution to narrative emergence.
- By design, MCA, Ψ, and ING enable agents not only to act, but to self-narrate their actions, producing explanations that are native to the architecture.

### 6.3 Meta-computational narratives as structural proposal

We propose a new class of interpretability: meta-computational narratives.

- These are recursive, self-descriptive accounts generated internally by the system.
- They function not primarily as user-facing heuristics, but as epistemic scaffolds supporting corrigibility, alignment, and transparency.
- Narrative reflexivity thus becomes an infrastructural property, rather than an optional diagnostic (Table 10).

**Table 10:** Summary of paradigmatic shift.

Dimension	Localist/Post-hoc	Modular/Topological	Narrative/Meta-computational
Temporal scale	Static snapshot	Episodic modular trace	Sequence-level, generative
Semantic resolution	Low (token/probability)	Medium (concept/modules)	High (storylines, agent modeling)
Operational Integration	External probe	Partially embedded	Natively embedded and reflexive
Cognitive alignment	Weak	Moderate	Strong

Compares three interpretability paradigms: localist/post-hoc, modular/topological, and narrative/meta-computational.

Having reframed interpretability as a meta-computational narrative, we now articulate the epistemological horizon this paradigm opens for future AI systems.

### 6.4 A call for a new epistemology of AI

Ultimately, this work is a call for a new epistemology of AI:

- One where interpretability is not applied externally, but designed into memory, planning, and updating mechanisms.
- One where agents generate reflexive interpretive loops as they evolve.
- One where explanation is co-equal with computation, not its by-product.

**Figure 12:**
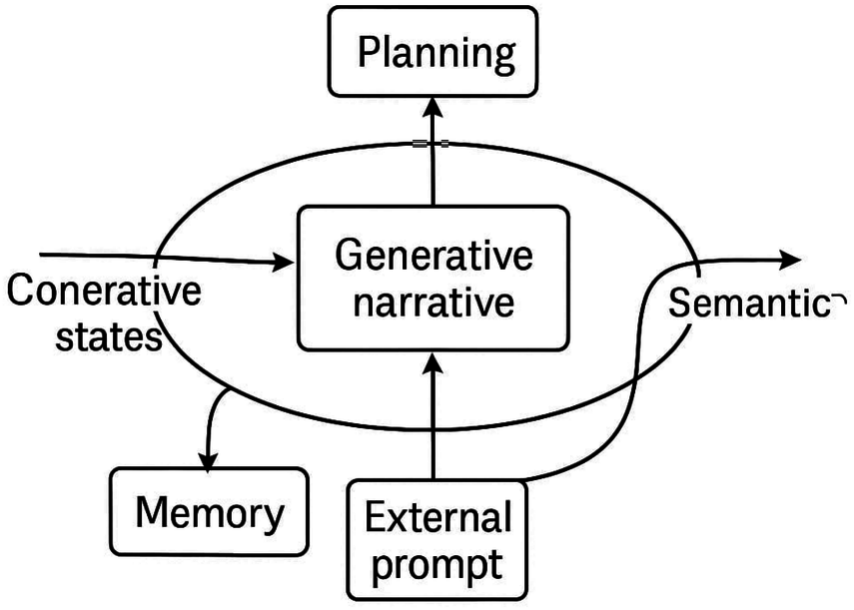
Schematic of the narrative interpretability loop. Conceptual diagram showing internal states feeding into MCA, Ψ, and ING, with arrows toward memory, planning, and external prompting. Highlights reflexivity and long-range coherence.

We term this vision a Science of Meta-Computational Narratives, in which understanding, explanation, and computation co-evolve as inseparable properties of intelligent systems (Figure 12).

## Data Availability

The raw data supporting the conclusions of this article will be made available by the authors, without undue reservation.
