# Supplementary material for: Epistemic limits of local interpretability in self-modulating cognitive architectures
Source: Front Artif Intell. 2025 Dec 1;8:1677528. doi: 10.3389/frai.2025.1677528 (PMC12702970; doi:10.3389/frai.2025.1677528)
Supplement: Supplementary file 1 [file Data_Sheet_1.docx]

**Appendix A: Procedural Annex: Reproducibility Framework:**

This appendix provides the complete procedural description required to reproduce the experiments described in Section 3. It includes simplified pseudo-code, random seed initialization, dataset descriptions, and repository organization guidelines.

**A.1 Pseudo-code for Core Modules:**

The following pseudo-code summarizes the key computational elements introduced in Section 3 (MCA, Ψ, ING).

Algorithm A1 — Modular Cognitive Attention (MCA)

def MCA (contexts, salience_vector, memory):

# contexts: list of local context nodes

# salience vector: σ values for each context

# memory: recursive contextual memory (RCM)

attention_scores = softmax(Wq @ contexts @ Wk.T)

modulated_scores = attention_scores * salience_vector

updated_contexts = attention_scores @ contexts + memory

return updated_contexts, modulated_scores

Algorithm A2 — Cognitive Leap Operator (Ψ)

def CognitiveLeap(ci, contexts, salience_threshold):

# ci: current context

# contexts: all other contexts

# salience_threshold: θ

candidates = [cj for cj in contexts if distance(ci, cj) >> 0]

for cj in candidates:

if gradient_salience(ci, cj) > salience_threshold:

return cj # perform leap

return ci # no leap triggered

Algorithm A3 — Internal Narrative Generator (ING)

def ING(contexts, trajectories):

# contexts: evolving cognitive states

# trajectories: historical sequence of states

narrative = []

for t in range(len(trajectories)):

coherence_score = measure_coherence(trajectories[:t])

narrative.append(generate_summary(contexts[t], coherence_score))

return narrative

**A.2 Random Seed Initialization:**

All experiments were conducted with fixed seeds for reproducibility:

Library Seed Example

NumPy 12345

PyTorch 42

Random (Python) 7

CUDA deterministic Enabled

Initialization snippet:

import numpy as np, torch, random

np.random.seed(12345)

torch.manual_seed(42)

random.seed(7)

torch.backends.cudnn.deterministic = True

torch.backends.cudnn.benchmark = False

**A.3 Simulated Datasets:**

Since real BMI/neurocognitive data could not be used for ethical reasons, we generated synthetic datasets with controlled parameters.

- Dataset A1: Context Graph Simulation:
- Format: CSV with 5,000 rows × 10 columns
- Columns: context_id (int)
- knowledge_vector (array, length 128)
- intention_score (float)
- reasoning_state (categorical: {deductive, analogical, narrative})
- salience (float, range 0–1)
- time_step (int)
- Dataset A2: Perturbation Protocol Logs:
- Format: JSON logs
- Contains each perturbation type (salience_injection, intent_noise, etc.) and observed metrics (leap_rate, emergent_nodes, coherence_score).
- Used to populate Table 5 in Section 3.4.

**A.4 Repository Structure:**

If uploaded, the repository would follow this structure :

/necap-simulations

│

├── data/

│ ├── dataset_A1_context_graph.csv

│ └── dataset_A2_perturbations.json

│

├── models/

│ ├── MCA.py

│ ├── CognitiveLeap.py

│ └── ING.py

│

├── experiments/

│ ├── run_experiment.py

│ ├── perturbation_tests.py

│ └── analysis_notebooks.ipynb

│

├── results/

│ ├── logs/

│ └── figures/

│

└── README.md

**A.5 Supplementary Visuals (to be generated):**

Figure A1: Pseudo-code Workflow Overview of MCA, Ψ, and ING modules in pseudo-code


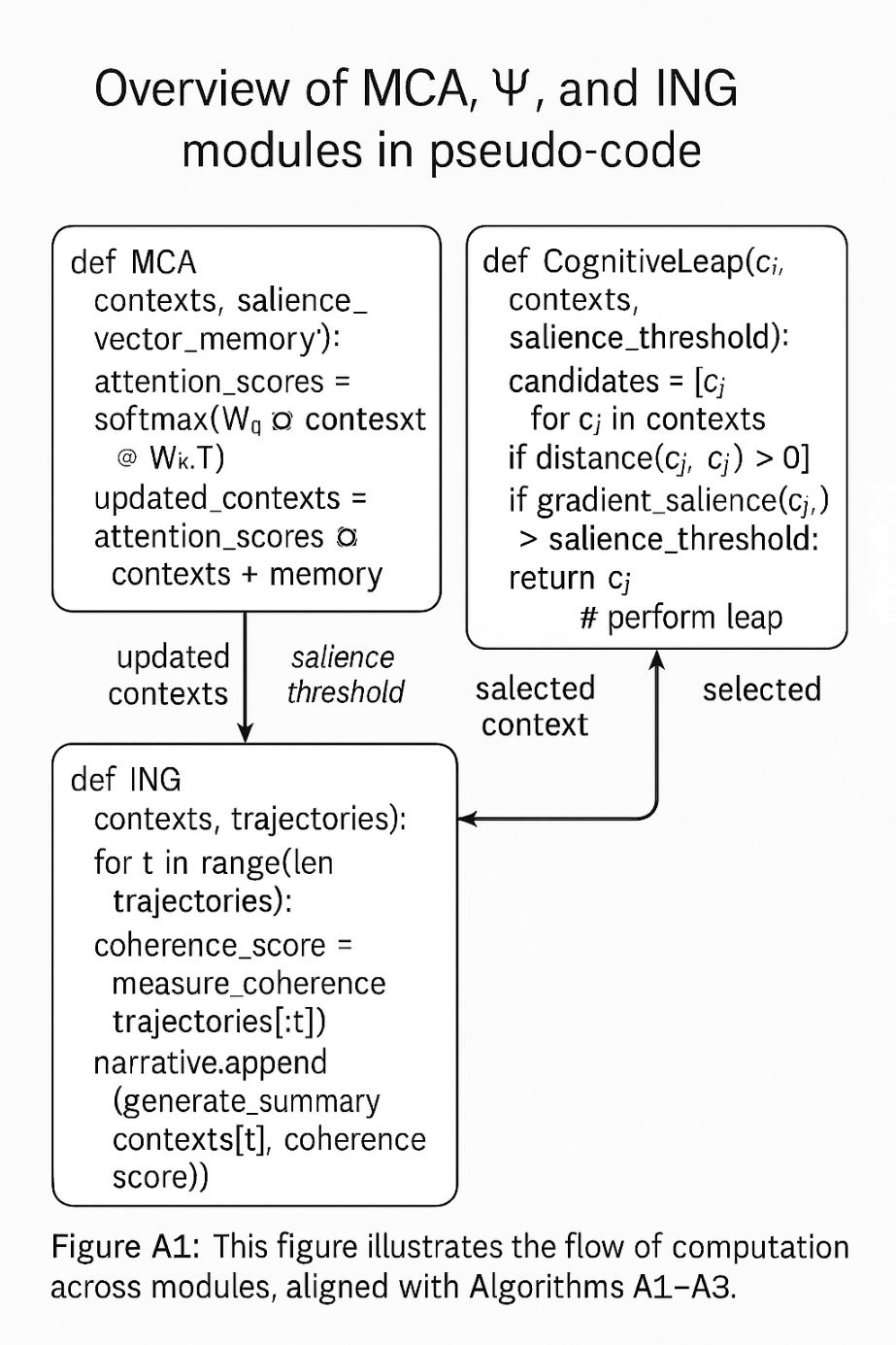


This figure illustrates the flow of computation across modules, aligned with Algorithms A1–A3.

Table A1: Random Seed Configuration: Seeds and deterministic settings for reproducibility

| Library / Component | Seed Value / Setting | Purpose / Effect |
| --- | --- | --- |
| NumPy | 12345 | Random number generation for arrays |
| PyTorch | 42 | Model initialization and training consistency |
| Python random | 7 | General-purpose randomness |
| CUDA deterministic | Enabled | Ensures deterministic GPU behavior |
| cuDNN benchmark | False | Disables auto-tuning for reproducibility |

Lists seed values and deterministic flags used to ensure reproducibility across experiments. These settings align with the initialization snippet provided in Section A.2 and guarantee consistent behavior across runs.

Figure A2: Dataset Structure: Simulated dataset schema (Context Graph and Perturbation Logs)


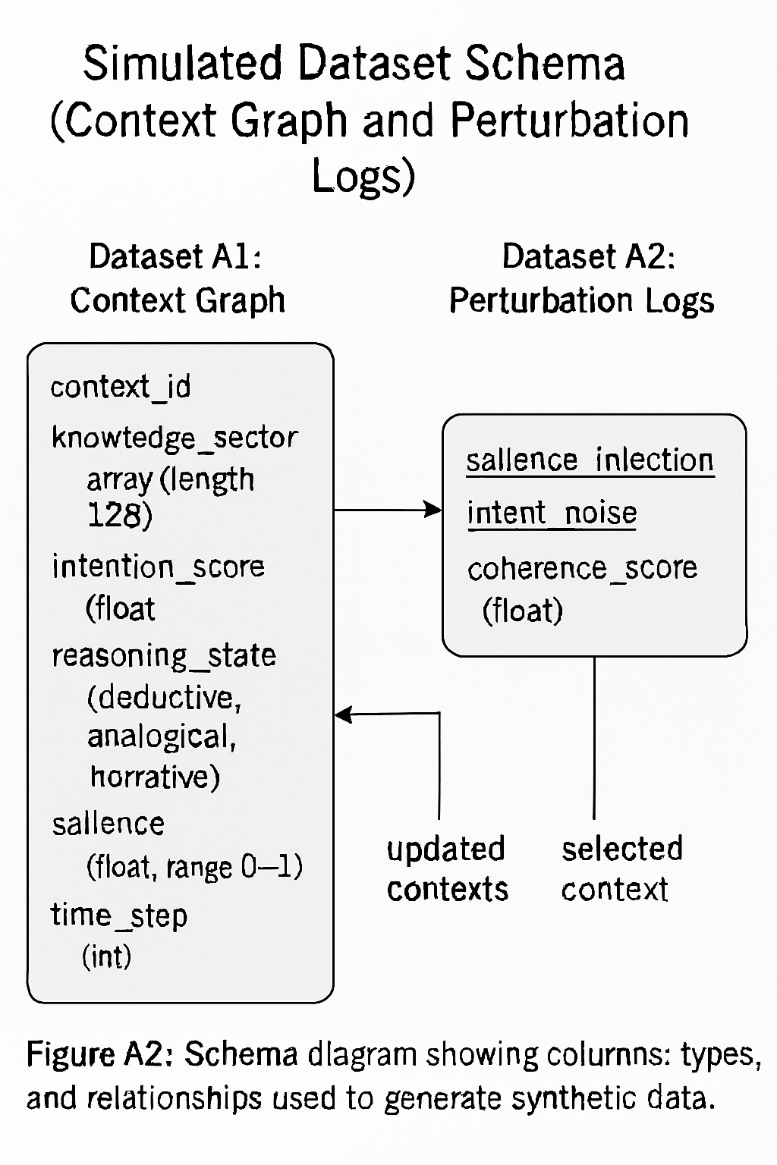


Schema diagram showing columns, types, and relationships used to generate synthetic data.

- Strategic Note:

This appendix complements Section 3 by making the framework replicable, transparent, and auditable. All core methods are expressed in pseudo-code, seeds are documented, dataset formats are explained, and repository organization is described. Together, these details ensure that external researchers could reproduce or extend the simulations without ambiguity.
